# Examining the Moderating Effect of Perceived Benefits of Maintaining Social Distance on E-learning Quality During COVID-19 Pandemic

**DOI:** 10.1177/0047239520977798

**Published:** 2021-06

**Authors:** Charu Saxena, Hasnan Baber, Pardeep Kumar

**Affiliations:** 1 University School of Business, 418665 Chandigarh University, Mohali, India; 2 Endicott College of International Studies, 41479 Woosong University, Daejeon, South Korea

**Keywords:** e-learning, social distancing, quality, learner, satisfaction, COVID-19, pandemic

## Abstract

Technology has influenced every aspect of our living, and education is not an exception. During the current pandemic period of COVID-19, the latent motive of maintaining social distancing is leading to be one of the prime reasons for the students to get enrolled in online courses. Although the benefits of e-learning have been discussed in various previous studies, it is important to understand the quality of e-learning and the satisfaction level of learners during this forceful shift toward e-learning amid the pandemic of COVID-19. This research proposes a conceptual model for understanding the variables influencing e-learning quality (ELQ) and learner satisfaction under the moderating effect of maintaining social distancing. The model is empirically validated by means of the partial least square approach through structural equation modeling based on 435 responses of university students in India. The results suggest that assurance, reliability, responsiveness, and website content are the factors that influence the ELQ of the online courses during the pandemic. ELQ also strongly influences the learner’s satisfaction. Interestingly, perceived benefits of maintaining social distancing have a significant negative moderating effect only between empathy and ELQ, which leads to the satisfaction of the learners.

E-learning refers to learning via the Internet, providing learners with a flexible and personalized platform to learn. It can be referred to be an innovative approach for an excellent provision of educational services to the learners through electronic information, aiming for continuous enhancement of their knowledge, skills, and other outcomes (Fazlollahtabar & Muhammadzadeh, 2012). It offers learning-on-demand possibilities and minimizes the learning cost (Zhang et al., 2008). E-learning is the evolution of distance and remote education—a learning situation where the instructor and learner are separated by distance, time, or both (Liaw, 2008). Recorded lectures by the instructors on online video streaming portals such as YouTube or on other websites are very popular among the students, especially to the ones who are learning through online education (Burke et al., 2009). A large number of universities and coaching institutions also provide a series of recorded lectures to the students. But to enhance the level of learning, it has become imperative now that instead of only listening or watching such lectures on the system, the learners should be effectively engaged from time to time by the program and the course through a variety of means such as assignments, quizzes, and discussion forums (Dixson, 2010). Further, increasing the opportunities for on-demand learning, in the light of intense interest in lifelong learning, is a noteworthy promise and potential of online learning programs (Akyol & Garrison, 2011; Kuraishy & Bokhari, 2009). Also, the students are able to actively choose among different massively available online certificate courses to address their professional and learning needs or to pursue personal interests, notwithstanding the presence of temporal, geographical, or institutional barriers (Adamopoulos, 2013). Moreover, online students are found to be self-assembling, mutual assisting, and utilizing online and in-person discussion groups (Bonvillian & Singer, 2013). The enrolled e-learning students perceived themselves to be more self-dependent and self-regulatory due to the inherent flexibility in the patterns of use of available study materials, the streaming of videos, assessment completion, and participation in the discussion forums (Campbell et al., 2014). The learning quality factors such as perceived usability, perceived value, and computer self-efficacy also have a significant impact on the satisfaction of such students (Isik, 2008). But, it is noteworthy to mention that students must have computer efficacy to ensure e-learning satisfaction (Roca et al., 2006).

Universities and higher education institutions, as the providers of the educational service, are striving hard to satisfy their customers, that is, the learners, through various students’ centric strategies and offerings (Martínez-Argüelles & Batalla-Busquets, 2016; Stodnick & Rogers, 2008). E-learning with lesser physical infrastructure costs, more variety of choices of courses and programs, larger integration with the global educational environment, and absolute freedom of place, time, and pace of learning are emerging as a great tool to serve this need of these educational service renderers. Further, during the present times of the COVID-19 pandemic period when the whole world is facing a health crisis and complete or partial lockdowns, the learners are enforced to pursue online courses for continuing their education (Baber, 2020). Universities and higher educational institutes worldwide are shifting toward various forms of online learning, and for the majority of them, it is an unchartered territory (Telles-Langdon, 2020). During these unprepared transitions, the educational institute administrators, faculties, and students are facing some abrupt unprecedented complications related to online learning (Moorhouse, 2020). Although any learning is aimed and directed to impart quality learning, through enhancing the learners’ satisfaction (Guragain, 2016), Lewnard & Lo (2020) stated that this transition during COVID-19 is forceful and unplanned; hence, the quality of learning and learner’s satisfaction emerges as the great point of research. 

Quality of an object has been defined as the “fitness for use” (Juran, 1981, pp. 15), conforming to requirements(Crosby, 1979), or absence of imperfections while satisfying the associated needs (Yang & Liu, 2007). To measure the quality of service, the most recognized quality measurement scales (SERVQUAL) are proposed by Parasuraman et al. (1988). Among the first studies to examine the quality of e-learning, Stodnick and Rogers (2008) found that only three SERVQUAL factors (assurance, empathy, and reliability) were true predictors of measuring the quality of e-learning and student satisfaction. In the addition to the SERVQUAL factors, some other variables such as web content and learning content were also tested to examine the e-learning quality (ELQ). “Web Content” refers to the use of the multimedia (audio, video, and graphics) nature of e-learning, as well as the utility, accuracy, and quality of the information found at the educational website (Udo et al., 2011). “Learning content” refers to available and correct learning material provided to students in an organized and timely fashion (Uppal et al., 2018). Learning content can range from the noninteractive course material, course quizzes, and case studies to highly collaborative, tailored or collective learning (Wu et al., 2012). Learning content quality further comprises the content richness and updates regularity (Lee & Lee, 2007). Learning content provided by the instructor enhances the perception of system usefulness and experience of e-learning (Lee et al., 2009).

Till now enough studies are undertaken to investigate the quality of e-learning, especially in the context of the developed world. But there are not many studies focusing on validating the developed world studies’ outcomes to the learners of the Indian sub-continent. Moreover, in the present COVID-19 times, the influence of maintaining social distancing, perceived harm of being on campus, and instead of taking online classes under lockdown may have altogether different implications on the quality of learning and learners’ perception of satisfaction thereto. Thus, the aim of the present study is to test the proposed hypothesized model using the partial least square structural equation modeling (PLS-SEM) technique to assess the impact of various e-learning factors under the moderating perceived impact of maintaining social distancing on the ELQ and its subsequent impact on the student satisfaction.

## Literature Review

Although the benefits of e-learning have been widely discussed in various previous studies, it is more critical now to better understand the satisfaction level of e-learners, especially as maintaining social distancing has become a new norm during this pandemic period. E-Learning is the delivery of education or training using electronic means or information technology to access the educational curriculum outside of a traditional classroom (Sangrà et al., 2012). Online courses and programs are being used more widely to augment or replace traditional classroom-based learning (Zhang et al., 2012). The current pandemic of COVID-19 and the purpose behind maintaining social distancing has led educational institutions at all levels to shift to e-learning. The capability to correctly assess the quality of e-learning is of great importance to all the stakeholders involved (Gress et al., 2010).

The SERVQUAL scale has been used in past to measure the service quality in various service industries such as banking (Savić & Veselinović, 2019), hospital (Pekkaya et al., 2019), hotel (Beheshtinia & Farzaneh Azad, 2019), automobile service (Baber, 2018), and education (Șerban & Stoian, 2019). The scale has been modified and tested in various online environment contexts, including e-learning (Ivanaj et al., 2019), e-banking (Baber, 2019), online shopping (Kim & Jackson, 2009), e-ticketing on airline websites (Elkhani et al., 2014), and so forth. Various studies have used this scale in online learning or e-learning environment (Sinclaire, 2011; Tan & Kek, 2004; Udo et al., 2011; Uppal et al., 2018). The factors of the SERVQUAL scale have been modified as per the context of the study and environment. The most common factors examined in online learning are assurance, empathy, reliability, responsiveness, learning content, and Website Content. 

The quality of e-learning may be understood better by various underlying theories and principles, including cognitive theory of multimedia learning, social cognitive theory, and information systems continuance model. The cognitive theory of multimedia learning (Mayer, 1997) established that individuals learn more intensely from pictures and words rather than from words alone. Visualization and audio have a greater role to play in learning, especially in e-learning where the “looks,” that is, website/App’s graphic design, layout, color, and fonts, and “feel,” that is, website/App’s identifiable, familiar features that help in navigation through the use of the interface, hyperlinks, and so forth, enhance learning outcome and lead to higher learning satisfaction. In this context, the website content is an essential differentiator for ELQ. Social cognitive theory (Bandura, 1986) endorses e-learner satisfaction spawning from successive interactions of a learner with the outside environment where the environment is already subjected to his cognition process before affecting the behavior. Behavior is affected by both cognitive factors and environmental factors (Wood & Bandura, 1989). Cognitive factors refer to the personal cognition beliefs and performance expectations of a learner, whereas environmental factors refer to the social and physical environments that can affect a learner’s behavior. According to the information systems continuance model, information system viability depends on its continued use and its continuance intention is determined by user satisfaction and perceived use. Further, the satisfaction of a user is dependent on the confirmation of expectations and perceived use. This underlines the importance of continued or repeated use of an e-learning platform by the e-learner to evidence the learning satisfaction. In the current COVID-19 pandemic times, there is a need to underdstand the perceived benefits of maintaining social distance or perceived threats of getting the deadly contagious disease by coming in touch with any COVID-19 positive person. Therefore, examining the moderating effect of such perception is important to understand the nature of online learning during the pandemic which may be different from online learning in absence of any crisis.

### Conceptual Framework and Hypotheses

**Figure 1.**
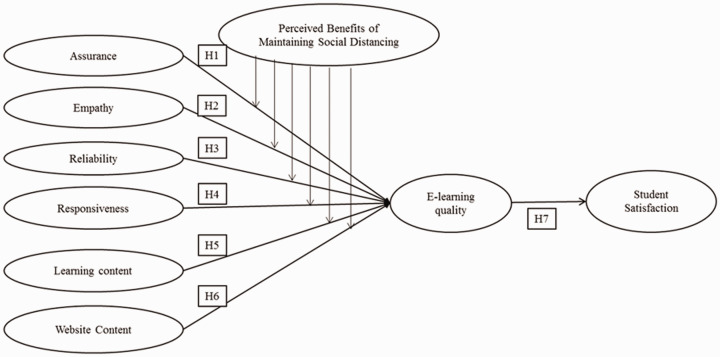
Hypothesized Research Model.

Based on the appraisal of previous significant studies and theories as discussed earlier, the researchers propose and empirically test a theoretical model (see Figure 1) that consists of six attributes of e-learning service quality, that is, Assurance, Empathy, Reliability, Responsiveness, Learning Content, and Website Content; learners’ satisfaction. The moderating effect of perceived benefits of maintaining social distance during the COVID-19 pandemic is also included. The literature on these constructs and attributes is as discussed later along with the formulation of relevant hypotheses.

Assurance is referred to as knowledge and courtesy of employees and their ability to inspire trust and confidence (Pham et al., 2019; Stodnick & Rogers, 2008). Quality assurance assumes that online course aims are brought into line with accreditation standards and that assessment outcomes are further enforced for continuous improvement to guarantee high-quality learning (Chapman & Henderson, 2010). Empathy includes caring and individualized attention that the service firm provides to its customers (Udo et al., 2011). *Reliability* is the ability to perform the promised service dependably and accurately (Lee et al., 2009). Responsiveness means readiness to help clients and give timely service (Uppal et al., 2018). The quality of e-learning also depends upon the learning content offered by different online courses and the attractiveness of the course website (Uppal et al., 2018). It implies that the quality of online courses depends upon the learning content and the course website content along with other dimensions of ELQ (Lu & Chiou, 2010). Learning content has been found to be positively related to ELQ (Lu & Chiou, 2010; Pham et al., 2019; Uppal et al., 2018). The blend of multimedia (audio, video, and graphics) can be used to supplement writing to improve the quality of website content (Koernig, 2003; Montoya-Weiss et al., 2003; Nitse et al., 2004). The “Website Content” dimension has been used in previous studies about ELQ and satisfaction of students (Cao et al., 2005; Santos, 2003; Udo et al., 2011). Stodnick and Rogers (2008) found assurance and student satisfaction positively related to each other. Udo et al. (2011) found assurance, empathy, responsiveness, and website content positively influencing the ELQ leading to student satisfaction. However, reliability was found insignificant in influencing ELQ. Uppal et al. (2018) found assurance, responsiveness, course website, and learning content, are positively associated with the ELQ. There is a positive association between ELQ and students’ satisfaction (Adel, 2017).

Therefore, we propose the following hypotheses:

*H1: Assurance has a positive influence on the ELQ.*

*H2: Empathy has a positive influence on the ELQ.*

*H3: Reliability has a positive influence on the ELQ.*

*H4: Responsiveness has a positive influence on the ELQ.*

*H5: Learning content has a positive influence on the ELQ.*

*H6: Website Content has a positive influence on the ELQ.*

*H7: ELQ has a positive influence on students’ satisfaction.*

### The Moderating Effect of Perceived Benefits of Maintaining Social Distance During the COVID-19 Pandemic

Various studies have found that during outbreaks of the pandemic, human behavior changes, such as maintaining social distancing, can have a significant effect on its spread (Maharaj & Kleczkowski, 2012; Poletti et al., 2012). Koo et al. (2020) suggested that social distancing must be prioritized to prevent the community spread of COVID-19 till a vaccine is developed. Lewnard and Lo (2020) suggested that politicians and administration of the state need to impose strict social distancing rules and does not discriminate against anyone from following this rule. Social distancing must be imposed centrally, by closing all educational institutes and workplaces and canceling all public events (Kleczkowski et al., 2015). Social distancing norm is an extremely useful strategy in the early stage of spread when vaccination is not available (Hollingsworth et al., 2011). Therefore, the moderating effect of the perceived importance of maintaining social distancing during this pandemic period on the SERVQUAL factors and ELQ will be examined in this study. To achieve this, Hypothesis H8 is proposed:

*H8a, b, c, d, e, f: The relationship between quality factors (assurance, empathy, reliability, responsiveness, learning content, and website content) and ELQ is moderated by the perceived benefits of maintaining social distance.*

## Method

### Data Collection and Instrument

The data are collected through a structured questionnaire obtaining responses of 435 undergraduate and graduate management students (international and national) in India. Data collection for this study is conducted using a questionnaire with 5-point Likert scales. An online version of the questionnaire was sent to the undergraduate and postgraduate students, accompanied by a cover letter. The data were collected through snowball sampling within our network and asked our network to forward it further. The questionnaire was shared with students in an online class in English. A conceptual model framework is proposed for understanding the relationship between ELQ and learners’ satisfaction moderated by perceived benefits of maintaining social distancing (PBMSD). Based on the proposed research framework as shown in Figure 1, a survey instrument administrated in English was designed from the previous studies to gather data to test the research hypotheses. The items that depict each of the four original constructs of SERVQUAL (assurance, empathy, reliability, and responsiveness), website content, ELQ, and student satisfaction are taken from the previous studies of Stodnick and Rogers (2008), Udo et al. (2011), and Uppal et al. (2018). The three items of PBMSD are adopted from Kleczkowski et al. (2015). We conduct an exploratory factor analysis by forcing to load all measurement items in one factor without any factor rotation. All the loadings were above the acceptable minimum level.

### Demographic Profile of Learners

Based on the demographic information in Table 1, the majority of learners (72.4%) belong to the age category of 22–25 years. Among the total respondents, 48.7% of students are male and the rest of 51.3% of students are females. Also, 51.5% of students are Indians, whereas 48.5% are international students of the university whose responses are recorded. Around 73% of students have enough experience as they have frequently used online learning. Further, 95.4% of the respondents have prior experience of using e-learning which makes this sample suitable for analyzing the moderated variable.

**Table 1.** Demographic Profile of Learners.

Category	Options	Frequency	Percentage
Age	18–22	90	20.7
	22–26	315	72.4
	26–30	25	5.7
	Older than 30	5	1.1
Gender	Male	212	48.7
	Female	123	51.3
Nationality	India	224	51.49
	Afghanistan	43	9.89
	South Africa	34	7.82
	Bangladesh	28	6.44
	Bhutan	36	8.28
	Namibia	18	4.14
	Nepal	39	8.97
	South Korea	13	2.99
Level of education	Undergraduate	90	20.7
	Postgraduate	345	79.3
Prior online learning experience	Never	20	4.6
	Sometimes	98	22.5
	Very Often	317	72.9

## Data Analysis and Results

### Measurement Model Assessments

The values of composite reliability, the average variance extracted (AVE), and Cronbach’s alpha values are as shown in Table 2. The values of composite reliability and Cronbach’s alpha for each construct are greater than the suggested minimum threshold limit of 0.7 (Bland & Altman, 1997), which means that the data collected are reliable. To check whether each item extracted the information relevant to the corresponding construct, factor loadings are estimated. All the values of factor loadings except SAT2 and SAT3 were meeting the minimum criteria of 0.7 (Hair et al., 2019). The item SAT3 was retained as the value is close to the threshold minimum value; however, SAT2 was deleted for further analysis. To check the validity of data, convergent validity-measurement was checked through the AVE, and all the values are above the minimum level of 0.5 (Hair et al., 2019).

**Table 2.** Reliability, Validity, and Measurement.

Construct source	Indicator	Survey questions	Factor loadings**	Alpha	CR	AVE
Assurance	ASSU1	The instructor is knowledgeable in his/her field.	0.871	.915	0.940	0.796
Udo et al. (2011)	ASSU2	The instructor is fair and impartial in grading.	0.899			
	ASSU3	The instructor answers all the questions thoroughly.	0.894			
	ASSU4	I am confident the instructor has an expert understanding of the material.	0.905			
Empathy	EMP1	The instructor is genuinely concerned about the students.	0.814	.858	0.904	0.704
	EMP2	The instructor understands the individual needs of students.	0.898			
Uppal & Gulliver (2018)	EMP3	The instructor has the student’s best long-term interests in mind.	0.745			
	EMP4	The instructor encourages and motivates students to do their best.	0.890			
Reliability	REL1	The instructor consistently provides good lectures.	0.838	.812	0.888	0.726
Uppal & Gulliver (2018)	REL2	The instructor is dependable.	0.868			
	REL3	The instructor reliably corrects information when needed.	0.851			
Responsiveness	RESP1	The instructor quickly and efficiently responds to student needs.	0.895	.895	0.934	0.826
Udo et al. (2011)	RESP2	The instructor is willing to go out of his or her way to help students.	0.925			
	RESP3	The instructor always welcomes student questions and comments.	0.907			
Learning content	LER1	The e-learning system provides me with sufficient learning content.	0.807	.856	0.913	0.779
Cheng (2012)	LER2	The e-learning system often provides updated information.	0.913			
	LER3	The e-learning system provides the learning content that I need.	0.923			
Website content	WEB1	The website uses audio and video elements properly.	0.868	.945	0.958	0.820
	WEB2	The website uses animations/graphics and multimedia features properly.	0.908			
Udo et al. (2011)	WEB3	The course website has relevant course information and learning material.	0.923			
	WEB4	The course website can be easily accessed and navigated.	0.924			
	WEB5	The website provides high-quality information.	0.902			
Perceived benefits of maintaining social distancing	PSD1	If I were to engage in social distancing (e.g., by avoiding public transport and social events), I would lessen my chance of developing an infectious disease.	0.763	.727	0.846	0.647
Kleczkowski et al., (2015)	PSD2	I am encouraged by engaging in social distancing during times of infectious disease because I feel it would be a necessity to do it.	0.823			
	PSD3	I feel confident in my ability to engage in social distancing during times of infectious disease.	0.826			
E-learning quality	ELQ1	The overall quality of the instruction I get from online learning is (poor–excellent).	0.793	.828	0.886	0.660
Udo et al. (2011)	ELQ2	The instructional website seems to be up to date.	0.843			
	ELQ3	The instructional website works well.	0.857			
	ELQ4	The instructional website has clear instruction.	0.753			
Student satisfaction	SAT1	Would you agree to say that “I am satisfied with my decision to enroll in the online classes?”	0.894	.795	0.852	0.601
Udo et al. (2011)	SAT2	Would you agree to say that “My choice to enroll in online classes was a wise one?”	0.539*			
	SAT3	Would you agree to say that “I think I did the right thing when I paid for online learning service?”	0.665			
	SAT4	Would you agree to say that “I feel that my experience with online learning has been enjoyable?”	0.935			

*Note*. CR = composite reliability; AVE = average variance extracted; ASSU = Assurance; EMP = Empathy; REL = Reliability; RESP = Responsiveness; LER = Learning Content; WEB = Website Content; ELQ = E-Learning Quality; SAT = Satisfaction.*Deleted for further analysis. **All factor loadings are statistically significant at a 5% level.

Further, the Fornell–Lacker criterion is used to assess discriminant validity. This method compares the square root of the AVE with the correlation of latent constructs (Hair et al., 2019). The values in bold in Table 3 show that the variance of the latent constructs for its own indicator is higher than that of other latent constructs (Fornell & Cha,1994). 

**Table 3.** Fornell–Lacker Criterion Results.

	ASSU	ELQ	EMP	LER	PBMSD	REL	RESP	SAT	WEB
ASSU	**0.892**								
ELQ	0.105	**0.813**							
EMP	0.085	0.396	**0.839**						
LER	–0.003	0.264	0.314	**0.882**					
PBMSD	0.019	0.499	0.318	0.181	**0.805**				
REL	0.050	0.625	0.369	0.233	0.276	**0.852**			
RESP	0.069	0.531	0.478	0.227	0.360	0.582	**0.909**		
SAT	0.034	0.393	0.172	0.065	0.269	0.236	0.208	**0.798**	
WEB	–0.106	0.186	0.238	0.290	0.160	0.077	0.093	0.102	**0.905**

*Note*. ASSU = Assurance; EMP = Empathy; RESP = Responsiveness; REL = Reliability; LER = Learning Content; WEB = Website Content; PBMSD = perceived benefits of maintaining social distancing; ELQ = E-Learning Quality; SAT = Satisfaction.

Heterotrait-Monotrait ratio criterion is also used to check the discriminant validity. From the results of the study, the values (in bold) in Table 4 are less than 0.85 which confirms the absence of any issues related to discriminant validity, according to the rule of thumb (Henseler et al., 2015).

**Table 4.** Heterotrait-Monotrait Ratio (HTMT).

	ASSU	ELQ	EMP	LER	PBMSD	REL	RESP	SAT
ASSU								
ELQ	**0.122**							
EMP	0.097	**0.467**						
LER	0.039	0.309	**0.374**					
PBMSD	0.051	0.641	0.400	**0.229**				
REL	0.067	0.758	0.437	0.284	**0.358**			
RESP	0.075	0.616	0.541	0.257	0.442	**0.684**		
SAT	0.068	0.417	0.166	0.086	0.330	0.232	**0.193**	
WEB	0.115	0.208	0.265	0.319	0.188	0.111	0.103	**0.106**

*Note*. ASSU = Assurance; EMP = Empathy; RESP = Responsiveness; REL = Reliability; LER = Learning Content; WEB = Website Content; PBMSD = perceived benefits of maintaining social distancing; ELQ = E-Learning Quality; SAT = Satisfaction.

### Goodness of Fit

On the basis of the comprehensive analysis of measurement models and structural model, it is concluded that both models are validated. Also, the results exhibit that the proposed theoretical model of this study has significant predictive relevance and explanatory power. Although PLS-SEM does not generate overall Goodness of Fit indices, *R*^2^ and standardized root mean square residual value is considered as the primary way to evaluate the explanatory power of the model (Henseler et al., 2016). However, considering the recommendations of Henseler et al., (2016), we have calculated standardized root mean square residual, which is found to be equal to 0.053, that is, less than 0.08, with Chi-square of 1465.153 and Normed Fit Index (NFI) as 0.842. So, the model proves a good fit as per the criterion proposed by Henseler et al. (2016). 

### Estimated Relationship

**Figure 2.**
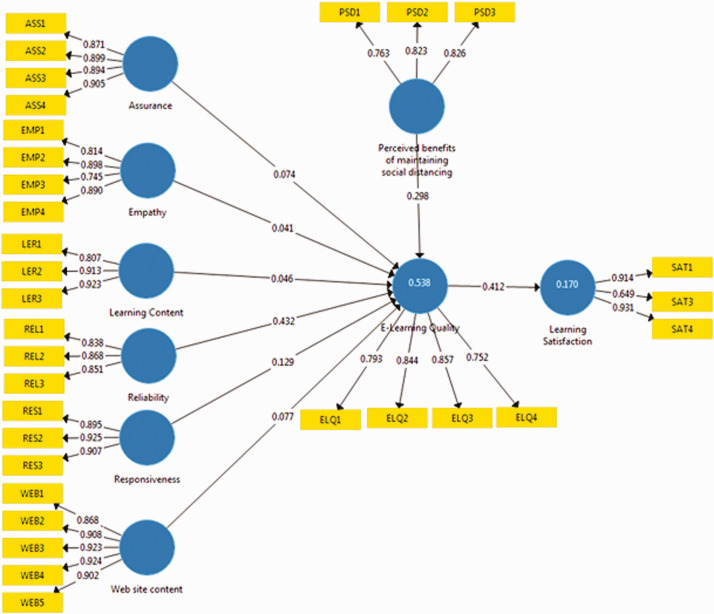
Structural Model of E-learning Quality and Satisfaction.

The standardized beta values (β) of path coefficients are computed by using the PLS algorithm function technique called bootstrapping (Hair et al., 2019) in SmartPLS 3.0. Assurance (β: .074, *p* < .05), Reliability (β: .432, *p* < .01), Responsiveness (β: .129, *p* < .05), and Website Content (β: .077, *p* < .05), have a significant positive effect on ELQ, whereas the empathy and learning content does not have a significant effect on ELQ. Further, ELQ (β: .412, *p* < .01), has a strong positive relationship with the learner’s satisfaction as shown in Table 5. Hypotheses H1, H3, H4, H6, and H7 are accepted; however, H2 and H5 are not supported. The *R*^2^ value of ELQ and learner’s satisfaction is .53 and .017, respectively. The path coefficient values and outer loadings of the item along with *R*^2^ values are shown in Figure 2.

**Table 5.** Path Coefficients.

Hypothesis	Path	Standardized beta	*t* Statistics	*p* Value	Result
H1	Assurance → E-Learning Quality	.074	2.457	.014	Supported
H2	Empathy → E-Learning Quality	.041	0.928	.354	Not supported
H3	Reliability → E-Learning Quality	.432	8.697	.000	Supported
H4	Responsiveness → E-Learning Quality	.129	2.194	.028	Supported
H5	Learning Content → E-Learning Quality	.046	1.322	.186	Not supported
H6	Website content → E-Learning Quality	.077	2.475	.014	Supported
H7	E-Learning Quality → Learning Satisfaction	.412	9.212	.000	Supported

### Moderating Effects of PBMSD

PLS-SEM bootstrapping procedure empirically measured the moderating effect of PBMSD on the relationship between various factors and ELQ. The bootstrapping results in Table 6 show that PBMSD significantly and negatively moderates the effect of empathy on ELQ (β = –.126, *p* < .05). This implies that high PBMSD can significantly reduce the effect of empathy on ELQ. PBMSD does not moderate the effect of any other variable on ELQ. Hence, the results confirm the acceptance of only one moderating hypothesis (H8b) and rejecting all other hypotheses. The empathy from faculty and staff of the educational institute during this period of the pandemic will not help students to enhance their learning and satisfaction rather it will reduce their satisfaction.

**Table 6.** Summary of the Moderating Effect of Perceived Benefits of Maintaining Social Distancing.

Hypothesis	Path	Standardized beta	*t* Values	*p* Value	Result
H8a	ASSU × PBMSD → E-Learning Quality	–.006	0.143	.886	Not supported
H8b	EMP × PBMSD → E-Learning Quality	–.126	2.906	.004	Supported
H8c	REL × LER → E-Learning Quality	–.030	0.572	.568	Not supported
H8d	RESP × PBMSD → E-Learning Quality	–.031	0.514	.607	Not supported
H8e	LER × PBMSD → E-Learning Quality	–.030	0.898	.369	Not supported
H8f	WEB × PBMSD → E-Learning Quality	.036	1.080	.280	Not supported

*Note*. ASSU = Assurance; EMP = Empathy; RESP = Responsiveness; REL = Reliability; LER = Learning Content; WEB = Website Content; PBMSD = perceived benefits of maintaining social distancing.

## Discussion

COVID-19 pandemic has disrupted almost all industries around the world, and the education sector is not an exception to it. Even continuing education during the pandemic, when social distancing norm is the only solution to slow down the spread, was a serious concern for all the educational institutes and learners. Most of the institutes, including schools and higher education, shifted toward online learning. Online learning is the best alternative available for continuing education. However, affordability, that is, inability to purchase electronic gadgets such as laptop, mobile, and pay for data, and so forth, and availability, that is, of internet connection and requisite infrastructure, is a matter of discussion among educators and policy makers. As the shift toward online education was sudden and somewhat forced, the quality of learning must not be compromised.

The study was aimed to check the influence of various factors of the SERVQUAL scale, the most common scale to assess the service quality and besides, other factors that are relevant to online learning particularly. As this online education is necessary to avoid COVID-19 spread on campuses, it is important to understand how students perceive the benefits of maintaining social distancing and its moderating effect on the various factors of ELQ. The factors such as assurance, reliability, responsiveness, and website content were found to be having a positive significant impact on ELQ, confirming previous similar investigations (Uppal et al., 2018) and, in turn, found to be having a strong relationship with learners’ satisfaction. The assurance factor explains that learners have the belief that their university administration and faculty are working hard, and they are assured they will get a quality education. Empathy toward learners during the pandemic in the online setup will not enhance the quality of learning. The reason may be that students expect universities to provide education for which they actually paid for not the empathy. Moreover, the interface is mostly impersonal in nature. Reliability is an important factor for students as they rely on their career and job prospects on university education and the same factor is true for online learning. The responsiveness variable may hold much importance during online education as students and instructors are placed in remote locations. The responsiveness of instructors and administration will help to enhance the quality of learning in the online environment during the time when learners are frustrated and need technical support. The learning content may not hold much importance in the immediate concern of the students, as they are struggling to cope up with the new learning setup and medium of learning and are more concerned about effective and nondisruptive utilization. Learners may feel a sense of frustration because of the lockdown, and sudden shifts toward this learning and the content of learning may be secondary to the quality of learning. The website content of educational institutes must be easy to navigate and provide relevant information during the pandemic as it is the only interface between the learner and institute at the time of the pandemic. The relevant information, learning material, and easiness to navigate will improve the quality of e-learning and enhance student satisfaction. The ELQ strongly influences learner satisfaction which means the quality of learning will enhance student satisfaction which is important in online learning during this sudden shift.

The moderating effect of PBMSD has been concluded to be significant only between “empathy” and ELQ. The learners acknowledge the perceived benefits of maintaining social distance and a high perception of maintaining social benefits will highly influence the effect of empathy on the ELQ. The prominence of the “empathy” attributes is justifiable pertaining to the impact of the current pandemic of the psychological state and behavior of the individuals (Murray & Schaller, 2012). COVID-19 has already been found to be influencing the social and daily lives of individuals, and they have been trying to protect themselves through various means (Wang et al., 2020; Woodside, 2020). In this state of affairs, the empathy extended during the e-learning delivery may act as a barrier toward learning and satisfaction. This may be understood to have a sort of frustrating impact on the individuals, who is continuously going through trauma, fear, and uncertainty resulted due to the current COVID-19 pandemic. The results suggest that instructors should refrain from shown extra empathy during the pandemic as students do somewhat not like to stay back in homes and take classes in the online environment that is forced on them is already annoying. Baber (in press) found that under the moderating effect of maintaining social distancing, social interaction does not increase the effectiveness of online learning, rather students give more importance to continuous learning and saving lives rather than socializing in the online setting.

### Theoretical Implications

The present study has noteworthy implications and benefits for the subject area as it has been able to provide and validate a broad framework for the quality and satisfaction of e-learning, especially in the context of the new normal due to the COVID-19 pandemic. This is one of the first research in the area of studying the students’ e-learning in the changed new normal of a COVID-19 pandemic. Therefore, this study contributed to the literature by developing a framework in response to the COVID-19 pandemic. The study reconfirms the role of ELQ attributes, that is, assurance, reliability, empathy, responsiveness, website content, and learning content toward the satisfaction of e-learners, during the crisis as well. The discovered moderating role of maintaining social distancing on the relationship between “empathy” and ELQ posits a great case for further study and theory development. Empathy helps us in recognizing, sharing, and reacting to the emotions of others. Empathy is essential in taking the perspective of the other person’s mental life (Freud, 1921). Cognitive empathy wherein comprehending nonjudgmentally the positive and negative experiences and mental states of others (Bošnjaković & Radionov, 2018) upholds the larger role played by empathy in the e-learning service rendering. The present finding of this significant influence is validated by the presence of changed mental and psychological state of minds of the people due to the unprecedented developments caused by the ongoing COVID-19 pandemic. This also poses for the theoretical establishment of this phenomenon through wide research and to conclude whether this change is a temporary or permanent one.

### Practical Implications

Along with the academic implications, the present research also has noteworthy practical implications. Particularly, the current study is vital for the higher education institutions to start or review their e-learning offerings during the present current COVID-19 pandemic times explicitly triggering a paradigm shift in the world of learning and education. The positive change is inevitable, and the study outcomes may be used to establish better systems not only for the pandemic period but also for future times. E-learning or blended learning are the areas to be explored further by the learning solution providers. The established names in the physical education got to prove their metal in this new playfield by taking cues from the findings of this and related studies, whereas smaller players have got a level playing chance and a golden opportunity to establish themselves as the leaders of the learning and education industry. Another critical present-day concern for governments, policy makers, and education service providers is to understand the antecedents to the e-learning and their relationship with learning satisfaction and formulate the relevant strategies in accordance with these findings. The significant moderating effect of PBMSD between 'empathy' and ELQ calls for immediate action toward the provision of empathetic service solutions for effective delivery of e-learning and larger satisfaction of the e-learners.

## Conclusion

With the onslaught of COVID-19, it has become difficult to teach students through traditional classrooms. The students are compelled to enroll in online courses. During this challenging time period, the present study has given key quality factors of e-learning that can be improved by the e-learning service providers, institutions, and organizers. The path analysis with a structured equation model verified that ELQ relates to learner satisfaction. The results of this suggest that ELQ is positively influenced by the e-learning variables viz., assurance, responsiveness, reliability, and website content. The ELQ is the construct that strongly influences the learners’ satisfaction. The PBMSD only moderate the relationship between empathy and ELQ. With the progress of online education programs and improvement in quality factors, we believe e-learning will have a very bright future among young millennials.
